# Upregulation of circ_0076684 in osteosarcoma facilitates malignant processes by mediating miRNAs/CUX1

**DOI:** 10.1186/s13018-024-04742-8

**Published:** 2024-04-24

**Authors:** Pengfei Cai, Xin Fu, Xiaofei Li, Wei Zhao

**Affiliations:** Department of Orthopeadics, Affiliated Jinhua Hospital, Zhejiang University School of Medicine, No. 365 Renmin East Road, Jinhua City, 321000 Zhejiang Province China

**Keywords:** Osteosarcoma, Circular RNAs, RUNX2, miRNAs, CUX1

## Abstract

Circular RNAs (circRNAs) are a newly appreciated type of endogenous noncoding RNAs that play vital roles in the development of various human cancers, including osteosarcoma (OS). In this study, we investigated three circRNAs (circ_0076684, circ_0003563, circ_0076691) from the RUNX Family Transcription Factor 2 (RUNX2) gene locus in OS. We found that the expression of circ_0076684, circ_0003563, circ_0076691, and RUNX2 mRNA is upregulated in OS, which is a consequence of CBX4-mediated transcriptional activation. Among these three RUNX2-circRNAs, only circ_0076684 is significantly associated with the clinical features and prognosis of OS patients. Functional experiments indicate that circ_0076684 promotes OS progression in vitro and in vivo. Circ_0076684 acts as a sponge for miR-370-3p, miR-140-3p, and miR-193a-5p, raising Cut Like Homeobox 1 (CUX1) expression by sponging these three miRNAs. Furthermore, we presented that circ_0076684 facilitates OS progression via CUX1. In conclusion, this study found that the expression of three circRNAs and RUNX2 mRNA from the RUNX2 gene locus is significantly upregulated in OS, as a result of CBX4-mediated transcriptional activation. Circ_0076684 raises CUX1 expression by sponging miR-370-3p, miR-140-3p, and miR-193a-5p, and facilitates OS progression via CUX1.

**Supplementary Information:**

The online version contains supplementary material available at 10.1186/s13018-024-04742-8.

## Background

Osteosarcoma (OS) is a prevalent and fatal primary bone malignancy that preferentially affects children and adolescents [1, 2]. Despite current advances in OS treatment, the overall survival rate of many patients remains low due to its potent invasiveness, metastasis, and high recurrence rate [3, 4]. The biology of OS, especially the molecular aspects underlying the initiation and progression of malignant processes, is poorly understood, limiting the development and application of effective biomarkers and therapeutic targets. Therefore, there is an urgent need to discover the molecular mechanisms involved in the development of OS.

Recently, circular RNAs (circRNAs) have emerged as an important type of endogenous noncoding RNAs, drawing increasing attention. CircRNAs are generated through back-splicing events of pre-mRNAs and formed by covalently closed loops without 5′ caps and 3′ poly (A) tails [5]. They can perform various molecular functions, with the best-characterized function being as miRNA sponges to inhibit miRNA functions and upregulate the expression of miRNA target genes [6]. Accumulating evidence has shown that circRNAs play crucial roles in the development of various human diseases, particularly cancers [7, 8]. In OS, circRNAs have been identified as clinical biomarkers and critical regulators in tumor growth, metastasis, and chemotherapy response, showing promise for research and application [9].

A previous study analyzed the expression profile of circRNAs in OS cell lines and a normal human osteoblast line (hFOB1.19) using next-generation RNA sequencing [10]. It was found that the expression of three circRNAs (circ_0076684, circ_0003563, circ_0076691) from the RUNX Family Transcription Factor 2 (RUNX2) gene locus was significantly upregulated in Saos2 cells. Numerous studies have demonstrated the importance of RUNX2 in bone development and its dysregulation in the pathogenesis of OS [11–14]. Furthermore, RUNX2 amplification in osteosarcoma has been confirmed through next-generation sequencing [15]. Additionally, a recent study revealed that overexpressed Chromobox homolog 4 (CBX4) in OS can recruit GCN5 to the RUNX2 promoter to activate transcription [16]. Based on the above theoretical bases, we consider that the high expression of RUNX2-circRNAs may also be a consequence of the transcriptional activation of RUNX2 gene. In consideration of the vital roles of RUNX2 gene in bone diseases including OS, we chose RUNX2-circRNAs as the research objects to further study their expression, function, and mechanism in OS.

## Methods

### Tissue samples

Thirty-eight pairs of OS tissue samples and adjacent non-tumor tissue (ANT) samples were harvested from OS patients who received surgery at Affiliated Jinhua Hospital, Zhejiang University School of Medicine. This research was approved by the ethics committee of the Affiliated Jinhua Hospital, Zhejiang University School of Medicine (JHSZXYY-2023-101-03) and conducted in accordance with the Declaration of Helsinki. The patients or their legal guardians have signed written informed consent to authorize the use of the samples.

### Cell culture and cell transfection

Human OS cell lines (MG63, Saos2, 143B and U2OS) and normal osteoblast cell line hFOB 1.19 were purchased from the American Type Culture Collection (ATCC, USA). OS cells were cultured in Dulbecco’s modified Eagle medium (DMEM, Gibco, USA) and hFOB1.19 cells were grown in DMEM/F-12 (Gibco), both supplemented with 10% fetal bovine serum (FBS). Overexpression plasmids, siRNAs, miRNA mimics and miRNA inhibitors were designed and synthesized by RiboBio (Guangzhou, China). Lipofectamine 3000 (Invitrogen, CA, USA) was used to perform transfection according to the manufacturers’ instructions.

### Quantitative real-time PCR (qRT-PCR)

Total RNA from tissue samples and cultured cells was extracted using TRIzol reagent (Invitrogen, USA) to measure the concentration and purity with NanoDrop 2000 spectrophotometer (Thermo Fisher Scientific, USA). Then, a PrimerScript RT reagent kit (TaKaRa, Japan) was employed to reversely transcribe the isolated RNA into cDNA. RT-qPCR analyses were performed using SYBR Premix Ex Taq II (TaKaRa). β-Actin was selected as the internal reference of circRNAs, and primers were listed in Additional file 1: Table S1. Expression of miRNAs was detected using the miDETECT A Track miRNA qPCR Kits (RiboBio) and normalized to U6 expression.

### Cell proliferation assays

The proliferation ability of OS cells was assessed by cell counting kit-8 (CCK-8) assays and 5-Ethynyl-2′-deoxyuridine (EdU) assays. For CCK-8 assays, OS cells were seeded into a 96-well and incubated for 24, 48, and 72 h. Then, 10 µL of CCK-8 solution (Dojindo, Japan) was added to each well. A spectrophotometer was employed to measure the absorbance at 450 nm. A Cell-Light EdU Apollo488 In Vitro Kit (RiboBio) was employed to conduct EdU assays. The DAPI reagent was used to stain nuclei. The images were captured by a fluorescence microscope (Olympus, Japan) and the DNA replication activity was evaluated by the EdU-positive rate.

### Cell migration and invasion assays

Cell migration and invasion were evaluated using wound-healing and Transwell assays, respectively. For wound-healing migration assays, monolayer cells seed in 6-well plates were scraped with a straight scratch by 200 μl pipette tips. Then, cells were cultured in DMEM medium with FBS for 24 h. The images were captured at 0 h and 24 h to calculate the relative migration ability of OS cells. The matrigel-precoated Transwell chambers (8 μm, Corning, USA) were employed to perform Transwell invasion assays. Cells were re-suspended in 200 μL serum-free DMEM medium and added in the upper chambers. The lower chambers were added with 500 μl DMEM medium containing 10% FBS. After culturing for 24 h, the invaded cells were fixed and dyed with 0.1% crystal violet. The images were captured by a microscope (Olympus) to calculate cell numbers.

### Animal experiments

BALB/c nude mice (male, four-week-old) were purchased from the Animal Center of Affiliated Jinhua Hospital, Zhejiang University School of Medicine. All animal experiments were approved by the Animal Ethics Committee of Affiliated Jinhua Hospital, Zhejiang University School of Medicine (2022JHSZXYY-SYLL150-P03). Four mice from each group were subcutaneously injected with OS cells stably circ_0076684 overexpressed or silenced by lentivirus. The tumor size was recorded once a week according to the formula: volume = width^2^ × length × 0.5. After four weeks, all the mice were sacrificed and the tumors were resected.

### RNA fluorescent in situ hybridization (FISH)

The subcellular localization of circ_0076684 was displayed using a RNA FISH kit (GenePharma, China) containing specific probes targeting the back-splicing site of circ_0076684. The DAPI reagent was used to stain nuclei. The images were captured by a fluorescence microscope (Olympus).

### RNA pull-down assay

A biotinylated probes specificly targeting the back-splicing site of circ_0076684 was designed and synthesized by GenePharma. Briefly, cell lysate was incubated with circ_0076684 probes, and then, streptavidin magnetic beads were used to capture the biotin-coupled RNA complex. The RNA pulled-down was extracted and analyzed by qRT-PCR assay.

### Dual-luciferase reporter assay

The sequences of circ_0076684 or the Cut Like Homeobox 1 (CUX1) 3′UTR, including wild-types (Wt) or mutant versions (Mut), were synthesized and inserted into a pMIR-Report Luciferase vector. Next, luciferase reporters were transfected into OS cells together with miRNA mimics or corresponding negative controls (NCs). Luciferase activity was measured with a Dual Luciferase Reporter Gene Assay Kit (Beyotime Biotechnology, China) after 48 h.

### Western blot analysis

Total protein was extracted from cells using RIPA buffer containing protease inhibitors. The protein was separated by SDS-PAGE and transferred onto PVDF membranes. After blocking, the membranes were incubated with primary antibodies for CUX1 (ab54583, Abcam, USA) and β-Actin overnight and secondary antibodies for 1 h. Protein bands were detected using an Ultra High Sensitivity ECL Kit (MedChemExpress, USA).

### Statistical analysis

The SPSS 20.0 software (IBM, USA) and GraphPad Prism 5 software (GraphPad Inc., USA) was used to perform statistical analysis. Data from at least three independently reduplicate experiments are shown as the means ± standard deviation (SD). Student’s t-test or one-way analysis of variance followed by Tukey’s test was used for comparisons of groups. *P*-values less than 0.05 were considered statistically significant.

## Results

### RUNX2-circRNAs and RUNX2 mRNA are upregulated in OS which is mediated by CBX4

**Fig. 1:**
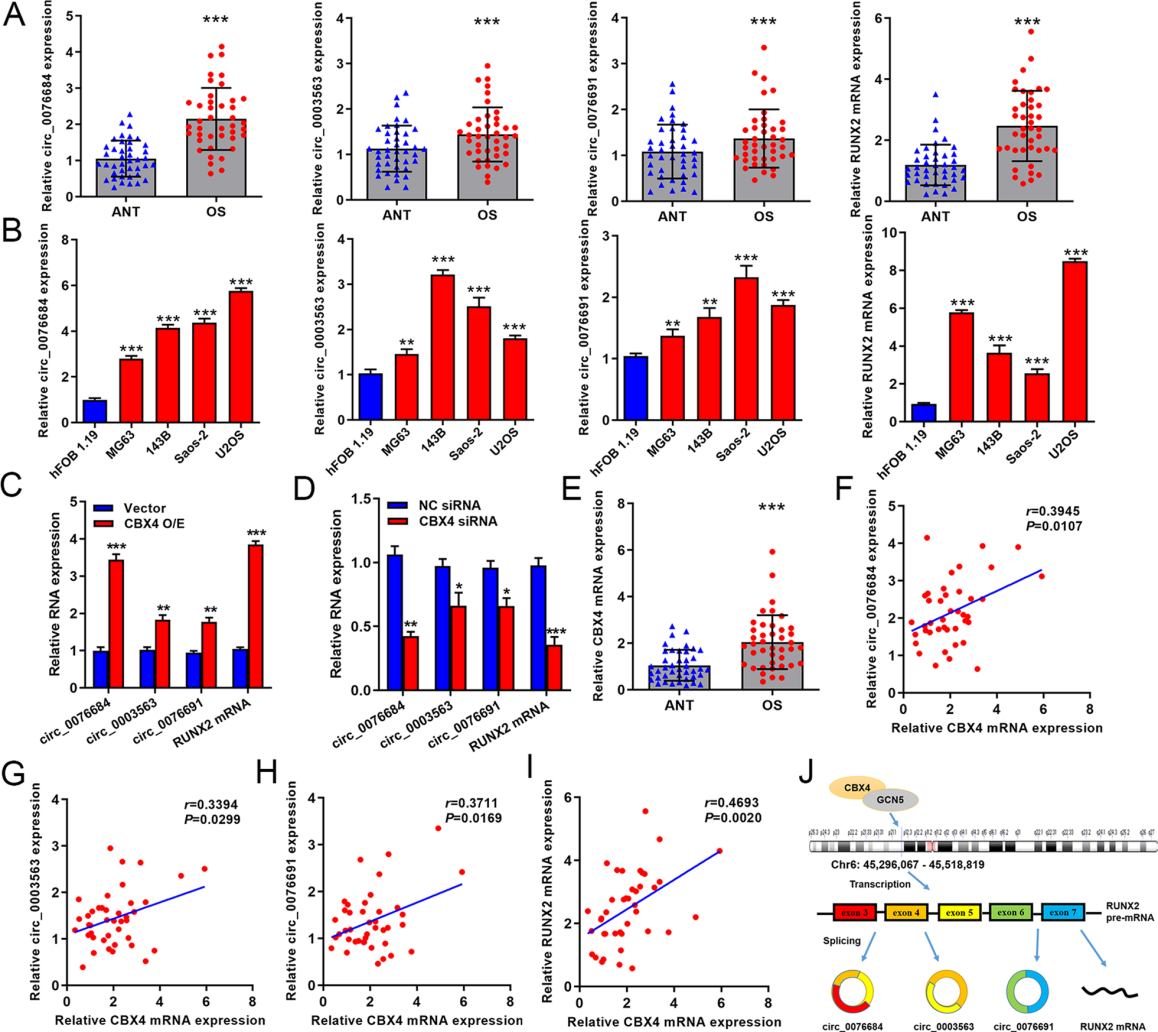
RUNX2-circRNAs and RUNX2 mRNA are upregulated in OS which is mediated by CBX4. **A** Expression of circ_0076684, circ_0003563, circ_0076691 and RUNX2 mRNA in OS tissues detected by qRT-PCR. **B** Expression of circ_0076684, circ_0003563, circ_0076691 and RUNX2 mRNA in OS cell lines detected by qRT-PCR. **C**, **D** Regulation of CBX4 on the expression of circ_0076684, circ_0003563, circ_0076691 and RUNX2 mRNA. **E** Expression of CBX4 mRNA in OS tissues detected by qRT-PCR. **F**, **I** Correlation of CBX4 mRNA with circ_0076684, circ_0003563, circ_0076691 or RUNX2 mRNA assessed by Pearson analysis. **J** Schematic diagram showing CBX4 mediating RUNX2-circRNAs and RUNX2 mRNA upregulation. ^*^P < 0.05, ^**^P < 0.01, ^***^P < 0.001

Firstly, we examined the expression of three circRNAs (circ_0076684, circ_0003563, circ_0076691) as well as RUNX2 mRNA from the RUNX2 gene locus. Our data revealed an increase in the expression of these RNAs in both OS tissues and cell lines (Fig. 1A, B), confirming the upregulation of RUNX2-circRNAs and RUNX2 mRNA in OS. Subsequently, we observed that the overexpression of CBX4 significantly enhanced the expression of circ_0076684, circ_0003563, circ_0076691, and RUNX2 mRNA, while knockdown of CBX4 decreased their expression (Fig. 1C, D). Additionally, CBX4 was upregulated in OS tissues and positively correlated with the expression of circ_0076684, circ_0003563, circ_0076691, and RUNX2 mRNA (Fig. 1E, I). Circ_0076684 consists of exons 3–5 in a loop structure, circ_0003563 consists of exons 4 and 5, and circ_0076691 consists of exons 6 and 7 (Fig. 1J). Based on previous studies stating that overexpressed CBX4 in OS can recruit GCN5 to the RUNX2 promoter to activate RUNX2 gene transcription [16], we concluded that the upregulation of RUNX2-circRNAs and RUNX2 mRNA is a result of CBX4-mediated transcriptional activation (Fig. 1J).

### Circ_0076684 significantly associates with clinical features and prognosis of OS patients

**Fig. 2:**
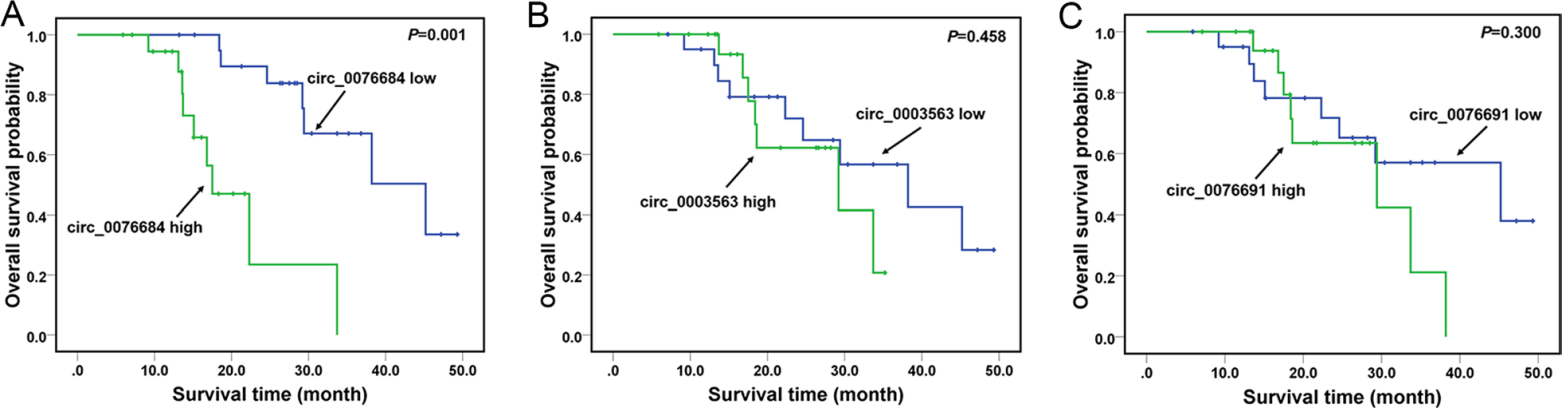
Circ_0076684 significantly associates with prognosis of OS patients. **A**–**C** Association of RUNX2-circRNAs with overall survival of OS patients was analyzed by Kaplan–Meier method

We then investigated the association of RUNX2-circRNAs with clinical characteristics and prognosis of OS patients. We found that circ_0076684 expression was significantly associated with tumor size, lung metastasis, Enneking stage, and overall survival time of OS patients (Table 1 and Fig. 2A). However, we did not observe any association between circ_0003563 or circ_0076691 with clinical characteristics and prognosis of OS patients (Table 1, Fig. 2B, C). These results suggest that circ_0076684, but not circ_0003563 or circ_0076691, may play a role in the progression of OS.

**Table 1:** Correlation between expression of RUNX2-circRNAs and clinicopathological characteristics of OS patients

Clinical variables (*N*)	circ_0076684 (low/high)	*P*	circ_0003563 (low/high)	*P*	circ_0076691 (low/high)	*P*
*Age*						
< 25y (24)	10/14		13/11		11/13	
≥ 25y (17)	11/6	0.128	8/9	0.448	10/7	0.308
*Gender*						
Male (22)	13/9		8/13		11/11	
Female (19)	8/11	0.220	12/7	0.134	10/9	0.558
*Tumor size*						
< 5 cm (25)	17/8		13/12		15/10	
≥ 5 cm (16)	4/12	0.008	8/8	0.577	6/10	0.139
*Lung metastasis*						
No (26)	17/9		15/11		15/11	
Yes (15)	4/11	0.019	6/9	0.222	6/9	0.222
*Enneking stage*						
I + IIA (25)	17/8		15/10		14/11	
IB + III (16)	4/12	0.008	6/10	0.139	7/9	0.328
*Location*						
Femur/tibia (28)	13/15		15/13		15/13	
Elsewhere (13)	8/5	0.287	6/7	0.457	6/7	0.457
*Differentiation*						
Well/moderately (27)	14/13		13/14		16/11	
Poorly/undifferentiated (14)	7/7	0.585	8/6	0.415	5/9	0.136

Data were analyzed by chi-square test

### Circ_0076684 promoted OS progression in vitro and in vivo

**Fig. 3:**
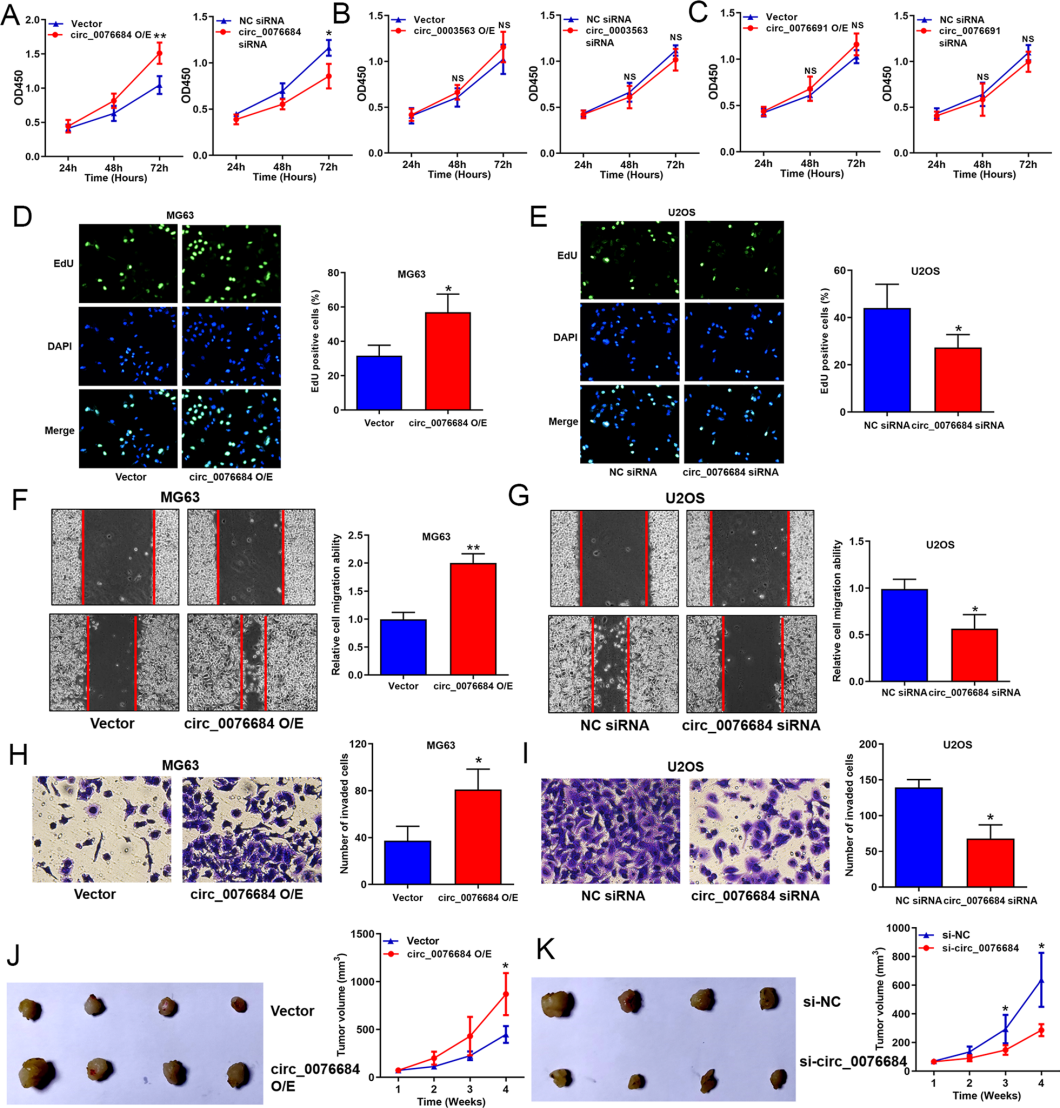
Circ_0076684 promoted OS progression in vitro and in vivo. **A**–**C** Regulation of circ_0076684, circ_0003563 and circ_0076691 on OS cell proliferation evaluated by CCK8 assays. **D**, **E** Regulation of circ_0076684 on OS cells proliferation evaluated by EdU assays. **F**–**I** Regulation of circ_0076684 on OS cell migration and invasion evaluated using wound-healing and Transwell assays, respectively. (J,K) Regulation of circ_0076684 on OS tumor growth in vivo evaluated by xenograft tumor model assays. ^*^P < 0.05, ^**^P < 0.01, ^***^P < 0.001

Subsequently, we explored the biological functions of RUNX2-circRNAs in OS. Initially, we overexpressed and silenced circ_0076684 in OS cells (Additional file 1: Fig. S1). CCK-8 assays indicated that among circ_0076684, circ_0003563, and circ_0076691, only circ_0076684 significantly promoted the proliferation of OS cells (Fig. 3A–C). Given the data in Table 1 and Fig. 2, we selected circ_0076684 for further analysis. EdU assays demonstrated that overexpression of circ_0076684 promoted proliferation, while silencing circ_0076684 inhibited proliferation of OS cells (Fig. 3D, E). Wound-healing and Transwell assays showed that overexpression of circ_0076684 enhanced migration and invasion, whereas silencing circ_0076684 suppressed these processes in OS cells (Fig. 3F–I). Xenograft tumor model assays revealed that overexpression of circ_0076684 promoted tumor growth, while silencing circ_0076684 inhibited tumor growth in vivo (Fig. 3J, K). These results collectively indicate that circ_0076684 promotes OS progression both in vitro and in vivo.

### Circ_0076684 acts as a miRNA sponge to raise CUX1 expression

**Fig. 4:**
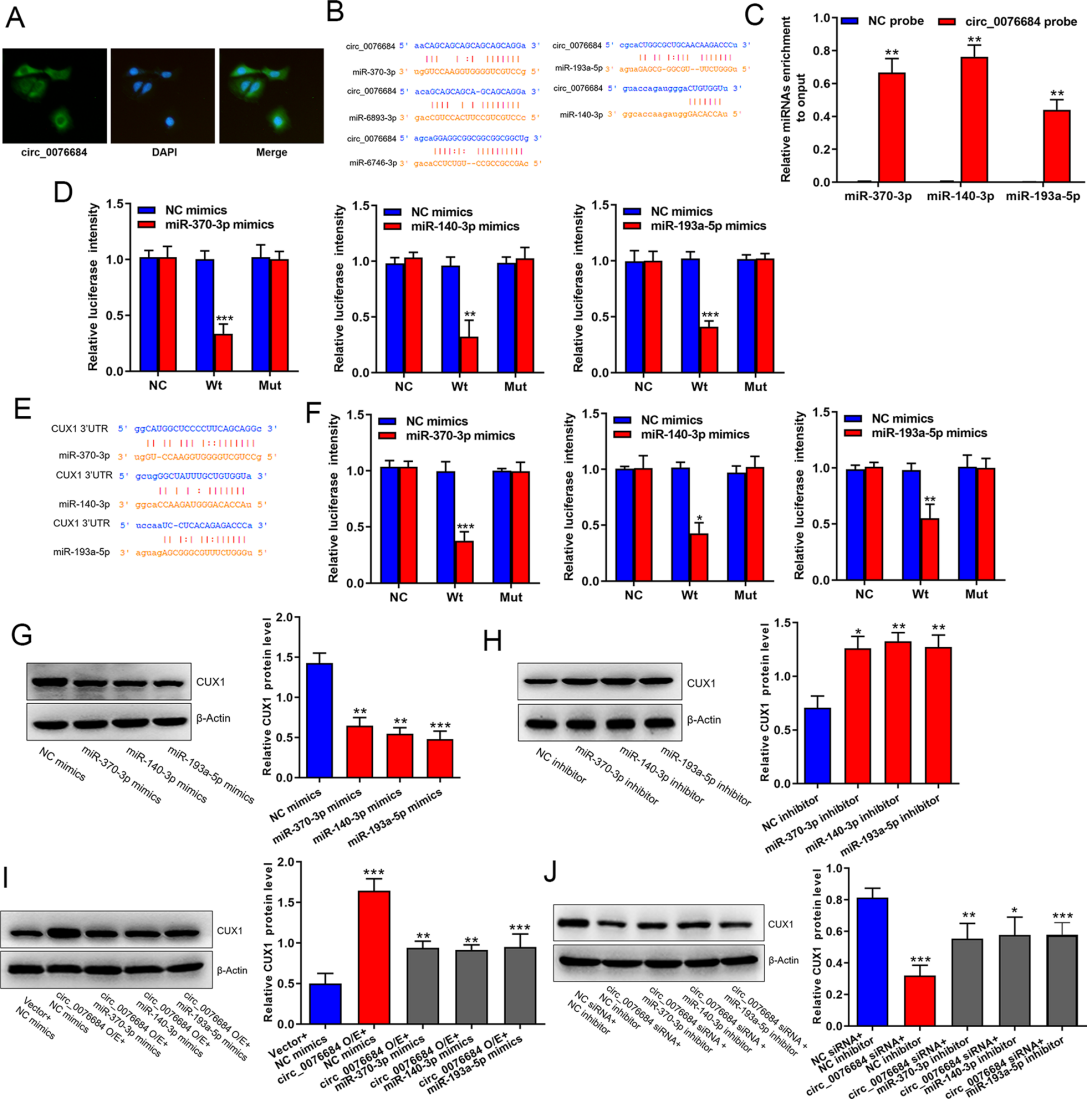
Circ_0076684 acts as a miRNA sponge to raise CUX1 expression. **A** Subcellular distribution of circ_0076684 detected by RNA FISH assay. **B** MiRNAs potentially binding to circ_0076684 predicted by Starbase database. **C** The interaction between circ_0076684 and miR-370-3p, miR-140-3p or miR-193a-3p validated by RNA pull-down assay. **D** The predicted binding sites of miR-370-3p, miR-140-3p and miR-193a-3p on circ_0076684 verified by dual-luciferase reporter assays. **E** Potentially binding sites of miR-370-3p, miR-140-3p and miR-193a-3p on CUX1 3′UTR predicted by Starbase database. **F** The predicted binding sites of miR-370-3p, miR-140-3p and miR-193a-3p on CUX1 3′UTR verified by dual-luciferase reporter assays. **G**, **H** Regulation of miR-370-3p, miR-140-3p and miR-193a-5p on CUX1 protein expression analyzed by western blot assays. **I**, **J** Regulation of circ_0076684 on CUX1 protein expression by miR-370-3p, miR-140-3p and miR-193a-5p analyzed by western blot assays. ^*^P < 0.05, ^**^P < 0.01, ^***^P < 0.001

Circ_0076684 comprises three exon elements and 627 nucleotides, surpassing both circ_0003563 and circ_0076691 in length, thereby demonstrating greater potential as a miRNA sponge. Using the RNA FISH assay, we discovered that circ_0076684 is predominantly located in the cytoplasm (Fig. 4A). Analysis through the Starbase database identified only five miRNAs (miR-370-3p, miR-6893-3p, miR-6746-3p, miR-193a-5p, and miR-140-3p) with predicted binding sites for circ_0076684 (Fig. 4B). Previous research has highlighted the cancer-suppressive roles of miR-370-3p, miR-140-3p, and miR-193a-3p in osteosarcoma [17–19], while the roles of miR-6893-3p and miR-6746-3p remain unexplored. Additionally, both miR-370-3p and miR-140-3p were predicted as target miRNAs for circ_0076684 by the Circular RNA Interactome database, leading to their selection for further investigation. Initial RNA pull-down assays confirmed significant enrichment of miR-370-3p, miR-140-3p, and miR-193a-5p in the RNAs pulled down by circ_0076684 probes (Fig. 4C). Subsequently, the predicted binding sites were validated through dual-luciferase reporter assays (Fig. 4D), indicating that circ_0076684 indeed acts as a sponge for these miRNAs.

Further analysis from the Starbase database revealed that miR-370-3p, miR-140-3p, and miR-193a-5p all target the same gene—CUX1 (Fig. 4E), a finding that was validated by dual-luciferase reporter assays (Fig. 4F). Additionally, CUX1 protein levels significantly decreased following the overexpression of these miRNAs and increased upon their silencing (Fig. 4G, H), confirming that CUX1 is a direct target of miR-370-3p, miR-140-3p, and miR-193a-5p. Moreover, overexpression of circ_0076684 led to an increase in CUX1 protein expression, while knockdown of circ_0076684 resulted in a decrease, effects that were mitigated by the overexpression and knockdown of miR-370-3p, miR-140-3p, and miR-193a-5p, respectively (Fig. 4I, J). Collectively, these findings demonstrate that circ_0076684 enhances CUX1 expression by sequestering miR-370-3p, miR-140-3p, and miR-193a-5p.

### Circ_0076684 facilitates OS progression via CUX1

**Fig. 5:**
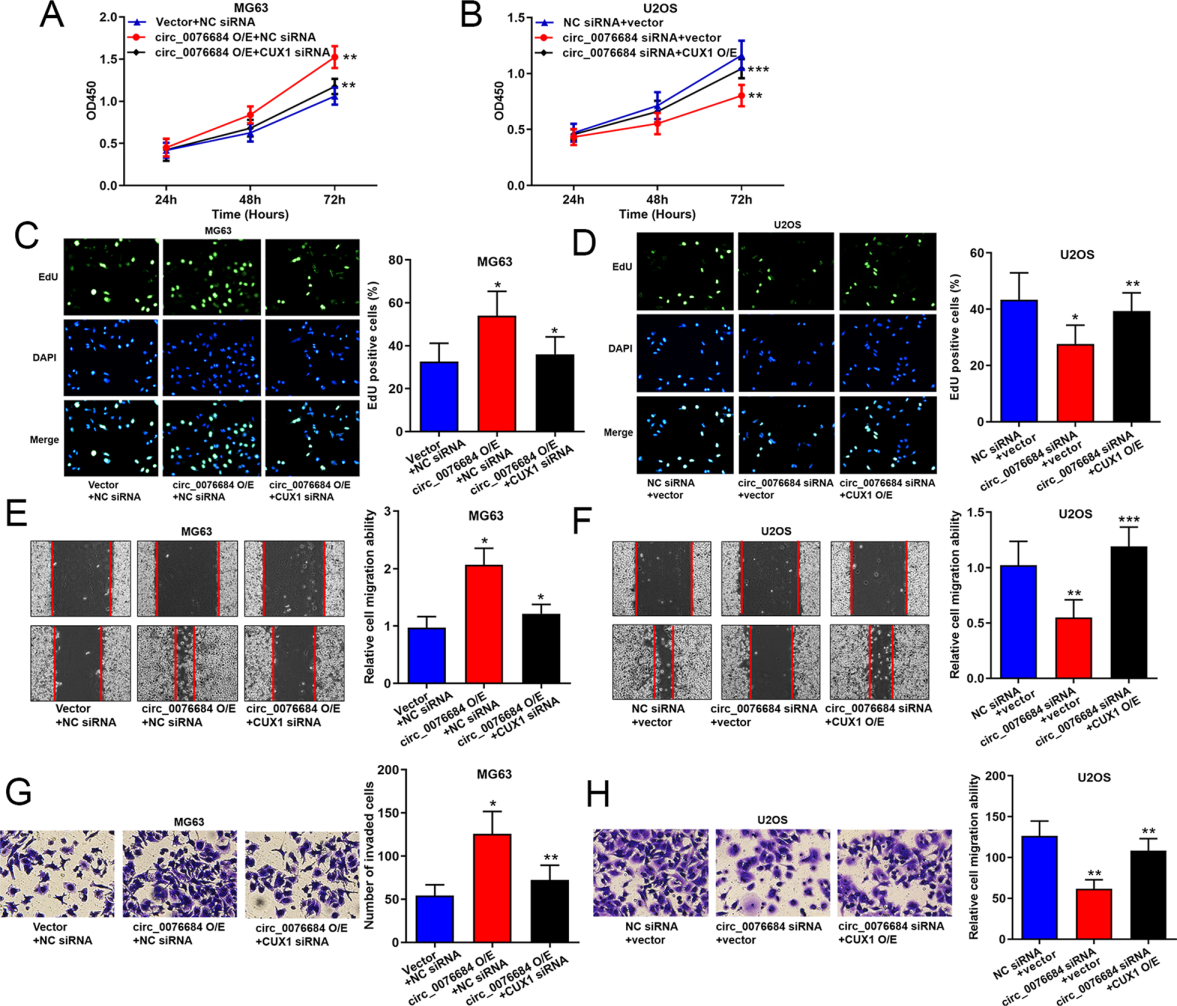
Circ_0076684 facilitates OS progression via CUX1. **A**–**D** Regulation of circ_0076684 on OS cells proliferation via CUX1 evaluated by CCK8 and EdU assays. **E**–**H** Regulation of circ_0076684 on OS cells migration and invasion via CUX1 evaluated by wound-healing and Transwell assays, respectively. ^*^P < 0.05, ^**^P < 0.01, ^***^P < 0.001

Given that circ_0076684 can upregulate CUX1 expression, we investigated the role of CUX1 in OS progression facilitated by circ_0076684. The CCK-8 and EdU assays demonstrated that CUX1 knockdown reversed the enhanced proliferation caused by circ_0076684 overexpression, while CUX1 overexpression mitigated the proliferation reduction resulting from circ_0076684 silencing (Fig. 5A–D). Additionally, wound-healing and Transwell assays revealed that CUX1 knockdown countered the increased migration and invasion driven by circ_0076684 overexpression, and CUX1 overexpression diminished the reduction in migration and invasion due to circ_0076684 silencing (Fig. 5E–H). These findings indicate that circ_0076684 accelerates OS progression through the modulation of CUX1.

## Discussion

Recent advancements in RNA sequencing and bioinformatics have led to the identification of numerous circRNAs that are dysregulated in various human cancers, including osteosarcoma (OS). In our study, we observed significant upregulation of three circRNAs (circ_0076684, circ_0003563, and circ_0076691) and RUNX2 mRNA from the RUNX2 gene locus in OS samples. We further established that the upregulation of RUNX2-associated circRNAs and RUNX2 mRNA results from CBX4-mediated transcriptional activation. While multiple studies have already confirmed the upregulation and oncogenic role of RUNX2 mRNA in OS [12–14], our research primarily focuses on the RUNX2-associated circRNAs for further investigation.

Among the three RUNX2-associated circRNAs analyzed, only the expression of circ_0076684 was found to be significantly correlated with crucial clinical parameters in osteosarcoma (OS) patients, including tumor size, lung metastasis, Enneking stage, and overall survival time. This discovery prompted a closer examination of the biological functions of RUNX2-circRNAs in OS. An initial functional assessment revealed that circ_0076684 notably enhanced OS cell proliferation, distinguishing it from the other circRNAs studied. These findings suggest that circ_0076684 plays a unique role in OS progression, leading us to focus our further research exclusively on this circRNA. Subsequent experiments confirmed that circ_0076684 significantly promotes proliferation, migration, and invasion of OS cells in vitro, as well as tumor growth in vivo. This evidence strengthens the association between high expression levels of circ_0076684 and the aggressive characteristics and adverse prognosis in OS, underscoring its potential as a key player in the disease's progression.

For the molecular mechanism underpinning its role in OS, circ_0076684, which comprises five exon elements and 627 nucleotides, stands out due to its length compared to circ_0003563 and circ_0076691, suggesting a heightened capability to act as a miRNA sponge. In this context, we found that circ_0076684 functions as a sponge for miR-370-3p, miR-140-3p, and miR-193a-5p. Given the established cancer-suppressive roles of miR-370-3p, miR-140-3p, and miR-193a-3p in OS, identified in previous studies [17–19], it is plausible that circ_0076684 may exert oncogenic effects by inhibiting the activity of these three miRNAs.

miRNAs usually regulate gene expression in a post-transcriptional manner via base-pairing with the 3’ untranslated regions (3’UTRs) of target mRNAs [20, 21]. In this study, our research demonstrates that CUX1, a gene implicated in various biological processes, including cell proliferation and differentiation, is a mutual target of miR-370-3p, miR-140-3p, and miR-193a-5p. Consequently, by sequestering these miRNAs, circ_0076684 effectively increases CUX1 expression, further elucidating its role in the progression of OS through this intricate molecular interplay.

CUX1 presents a paradox in cancer research, embodying dual roles that complicate its classification as solely oncogenic or tumor-suppressive. Some studies have found that diminished CUX1 expression is implicated in cancer development, suggesting a protective role when fully expressed [22–24]. Conversely, evidence also indicates that elevated CUX1 expression can advance cancer progression, underscoring its complex involvement in oncogenesis [22–24]. This study marks the first to elucidate the oncogenic function of CUX1 within the context of OS cells, revealing that silencing CUX1 can mitigate the cancer-promoting effects attributed to circ_0076684. Therefore, we conclude that circ_0076684 contributes to OS progression through its regulatory effect on CUX1 expression, highlighting a novel aspect of CUX1's multifaceted role in cancer biology. Given the potential application of RNA interference technology in human diseases in recent years [25–27], targeting circ_0076684, CUX1, and RUNX2 may have potential application value in the treatment of OS.

## Conclusions

**Fig. 6:**
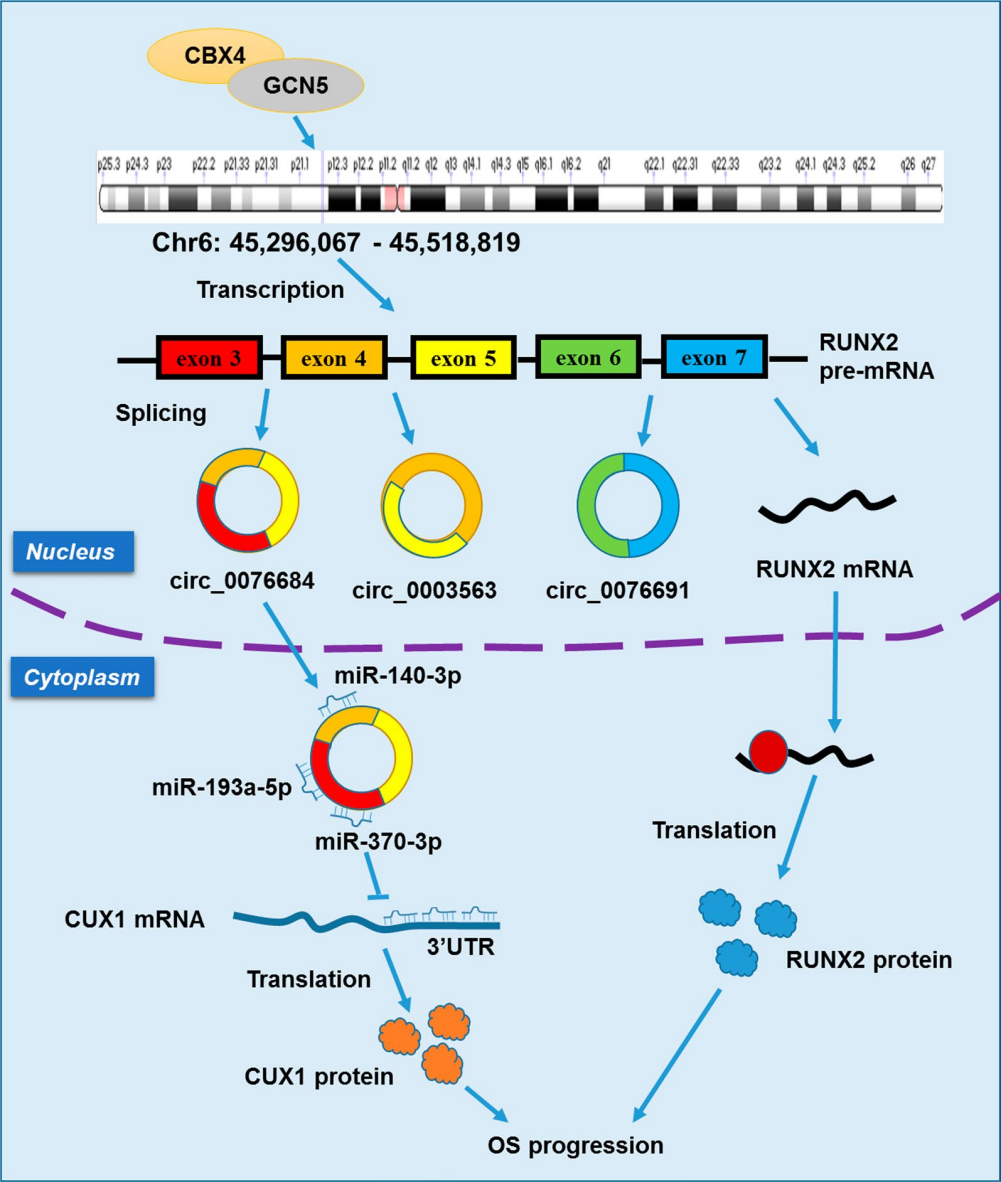
Diagram of RUNX2 gene locus in regulating OS progression

To sum up, this study found that the expression of three circRNAs (circ_0076684, circ_0003563, circ_0076691) and RUNX2 mRNA from RUNX2 gene locus is significantly upregulated in OS, which is a consequence of CBX4 mediated transcriptional activation. Circ_0076684 raises CUX1 expression by sponging miR-370-3p, miR-140-3p and miR-193a-5p, and facilitates OS progression via CUX1 (Fig. 6).

### Supplementary Information

**Additional file 1. Table S1. **Sequences used in this study. **Figure S1**. Overexpressing and silencing efficiency of circ_0076684, circ_0003563, and circ_0076691 in OS cells detected by qRT-PCR assay. ^*^P < 0.05,^**^P < 0.01,^***^P < 0.001.

## Data Availability

The datasets used and/or analyzed during the current study are available from the corresponding author on reasonable request.

## Abbreviations

circRNAs: Circular RNAs
OS: Osteosarcoma
RUNX2: RUNX Family Transcription Factor 2
CUX1: Cut Like Homeobox 1
CBX4: Chromobox homolog 4
ANT: Adjacent non-tumor tissue
qRT-PCR: Quantitative real-time PCR
CCK-8: Cell Counting Kit-8
FISH: RNA fluorescent in situ hybridization
3′UTRs: 3′ Untranslated regions
