# Supplementary material for: Upregulation of circ_0076684 in osteosarcoma facilitates malignant processes by mediating miRNAs/CUX1
Source: J Orthop Surg Res. 2024 Apr 24;19:260. doi: 10.1186/s13018-024-04742-8 (PMC11044396; doi:10.1186/s13018-024-04742-8)
Supplement: Supplementary file 1 — Additional file 1. Table S1. Sequences used in this study. Figure S1. Overexpressing and silencing efficiency of circ_0076684, circ_0003563, and circ_0076691 in OS cells detected by qRT-PCR assay. *P < 0.05,**P < 0.01,***P < 0.001. [file 13018_2024_4742_MOESM1_ESM.docx]

Table S1. Sequences used in this study

| Primers or siRNAs | Sequence (5’-3’) |
| --- | --- |
| circ_0076684 siRNA sense | CUCGGGAACCCAGAAAUCCGAdTdT |
| circ_0076684 siRNA antisense | UCGGAUUUCUGGGUUCCCGAGdTdT |
| circ_0003563 siRNA sense | CUCGGGAACCCAGAAGUGGUAdTdT |
| circ_0003563 siRNA antisense | UACCACUUCUGGGUUCCCGAGdTdT |
| circ_0076691 siRNA sense | AGGCGCAUUUCAGGGCACAGAdTdT |
| circ_0076691 siRNA antisense | UCUGUGCCCUGAAAUGCGCCUdTdT |
| circ_0076684 forward primer | CCGTCTTCACAAATCCTCCCC |
| circ_0076684 reverse primer | GCTCACGTCGCTCATTTTGC |
| circ_0003563 forward primer | CGTCTTCACAAATCCTCCCCA |
| circ_0003563 reverse primer | CATTCCGGAGCTCAGCAGAA |
| circ_0076691 forward primer | CTCTACCACCCCGCTGTCTT |
| circ_0076691 reverse primer | TGAGGAATGCGCCCTAAATCA |
| RUNX2 mRNA forward primer | CCGCCTCAGTGATTTAGGGC |
| RUNX2 mRNA reverse primer | GGGTCTGTAATCTGACTCTGTCC |


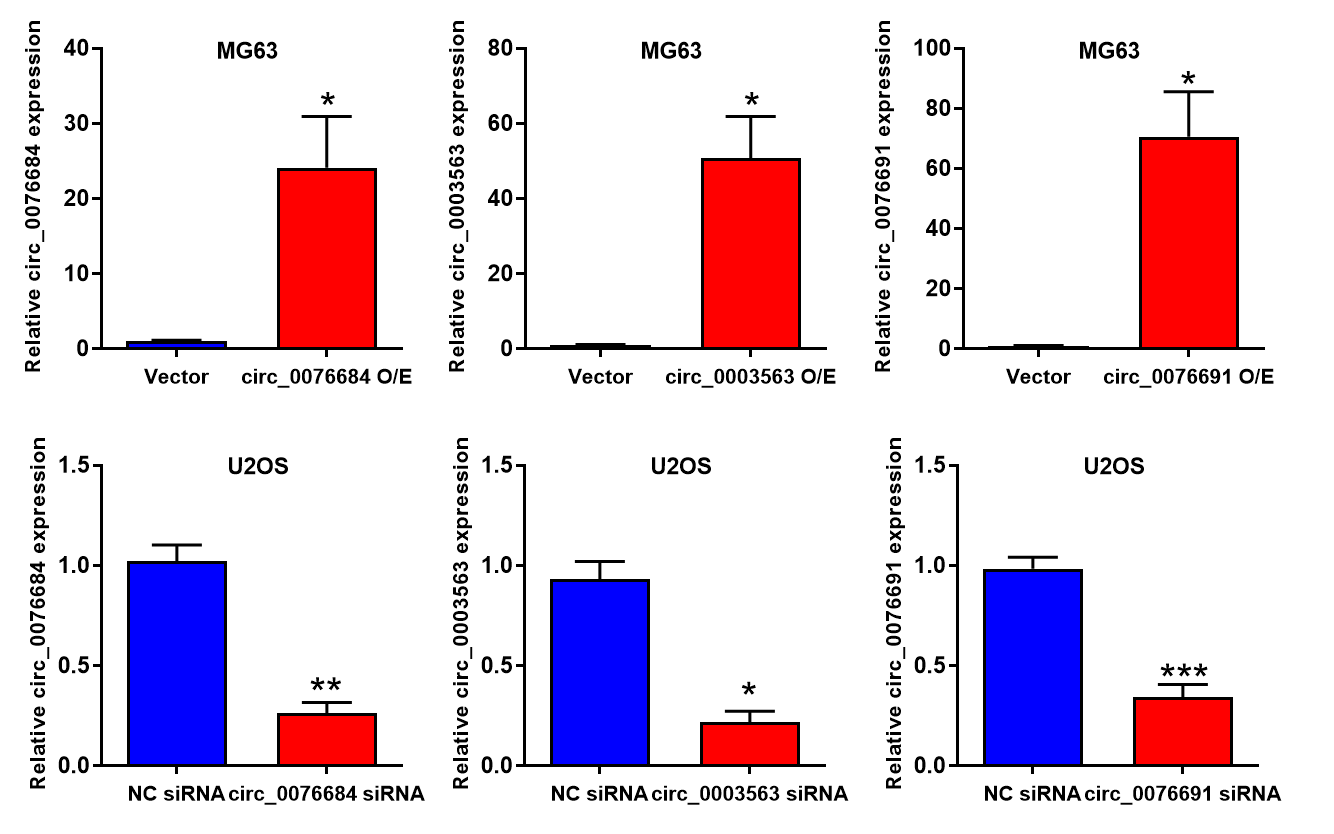


Figure S1. Overexpressing and silencing efficiency of circ_0076684, circ_0003563, and circ_0076691 in OS cells detected by qRT-PCR assay. ^*^*P*<0.05, ^**^*P*<0.01, ^***^*P*<0.001.
